# In invasion assays, the breast cancer cell nucleus leads the way

**DOI:** 10.1186/s13104-020-05314-9

**Published:** 2020-10-12

**Authors:** Malte Renz

**Affiliations:** Division of Gynecologic Oncology, Department of Obstetrics and Gynecology, Stanford University School of Medicine, 300 Pasteur Drive, H302, Stanford, CA 94305 USA

**Keywords:** Cancer cell invasion, Cancer cell metastasis, Cancer cell nucleus

## Abstract

**Objective:**

Cancer cell metastasis determines disease prognosis. During cancer cell metastasis, the cancer cell and the cancer cell nucleus have to undergo extreme shape changes. To monitor shape changes of cancer cells and cancer cell nuclei and the positioning of the cancer cell nucleus during cancer cell invasion, a customized invasion assay with 8-μm pores and reconstituted basal membrane was imaged using fluorescence live-cell microscopy.

**Results:**

The observed cells changed their shape from a distinct fibroblast-like spindle shape to an amoeboid shape without polarization immediately after the passage through an 8-μm pore of the invasion assay. During the process of invasion, the cancer cell centered the cancer cell nucleus over the 8-μm pore, and cancer cell nucleus and adjacent cytoplasmic areas moved first through such a pore. Seemingly testing if the largest and least deformable organelle may fit, the cancer cell nucleus led the way through the porous membrane of the invasion assay.

## Introduction

To spread to distant sites, cancer cells have to detach from their original cellular unit, squeeze through basal membrane and fibers of the extracellular matrix and find their way to adjacent blood or lymph vessels to be then distributed by the blood or lymph stream [1–3]. During this process of metastasis, cancer cells encounter narrow spaces of only a few micrometers in size that require the deformation of the entire cell [4]. In general, different features of cell locomotion have been described, including mesenchymal, lobopodial, and amoeboid locomotion. These different locomotion types may depend upon the dimensionality of the surrounding environment, the proteolytic activity of the migrating cells, and the extent of cell adhesions connecting a cell to the extracellular matrix [5, 6]. In immune cells, a differential positioning of the cell nucleus during locomotion has been shown [7, 8]. In a recent publication, Sixt and co-workers described how specific immune cells, i.e. dendritic cells, use their cell nucleus to probe the surrounding environment to find an appropriate pore size so that the cell nucleus and thus the entire cell can pass [8]. A similar positioning and role of the cell nucleus have not been reported yet in cancer cells [5, 8]. The recent study by the Sixt group on immune cells prompted me to review thus-far unpublished results of cancer cells that I collected during my doctorate in the 2000s. Here, I show observational evidence that (i) cells of a breast cancer cell line are able to change shape during cancer cell invasion in-vitro and that (ii) their cancer cell nucleus is leading the way in this process*.*

## Main Text

### Methods

#### Cell culture and plasmid

MDA-MB-231 cells (ATCC HTB-26, a human breast cancer cell line) were cultured in Dulbecco’s modified Eagle’s media (DMEM) supplemented with 10% fetal bovine serum (FBS) and 1% glutamic acid but without phenol red.

The coding sequence of the human CapG gene was amplified by polymerase chain reaction (PCR) with the primers (Invitrogen): 5′-TCG AGC TCA AGC TTC GAA TTC GGC-3′ and 5′-TAA TAA CCG CGG TTT CCA GTC CTT GAA AAA TT-3′. The amplified fragment was digested with EcoRI and SacII and inserted into the EcoRI and SacII sites of the pSV-eGFP vector (BD Biosciences Clontech, Heidelberg, Germany). The obtained construct was sequenced as described previously [9].

Using Transfectin (Biorad, Hercules, CA), the MDA-MB-231 cells were transfected with the pSV-CapG-eGFP construct. Stable cell lines expressing CapG-eGFP were established with neomycin/ G418 for selection.

#### Invasion assay

24-well format cell culture inserts (Corning, Biocoat Matrigel Invasion Chamber) with 8-μm pores and Matrigel, a basement membrane preparation, were used as invasion assays according to the manufacturer’s instructions. In brief, 24-well inserts were rehydrated for 2 h. 25,000 cells were suspended in Dulbecco’s modified Eagle’s cell culture media and seeded into the 24-well insert which was placed into the companion plate. The companion plate contained cell culture media with 10% fetal bovine serum and 200 ng/ml epidermal growth factor (Sigma) acting as chemotactic agents. The invasion assay was incubated at 37˚C, 5% CO2 atmosphere. Imaging experiments were performed at 37˚C in Hepes-buffered medium (pH = 7.4) 6–8 and 16 h after seeding, respectively.

#### Epifluorescence and confocal microscopy

MDA-MB-231 cells expressing CapG-eGFP were examined live using an Olympus CK40 epifluorescence microscope. Images were captured using an Optronis VX45 camera.

A laser-scanning confocal microscope (Nikon C1Si) with a 25-mW Argon ion laser was used to perform the imaging experiments with a 25 × 0.75 N.A. water objective. To move the porous membrane into the working distance of the objective, the inserts were placed on small sterilized plastic rings within a Lab-Tek chamber from Nunc. The confocal microscope was used to perform z-stack imaging. With the software Fiji the z-stacks were converted into movies and 3-D projections.

### Results

**Fig. 1:**
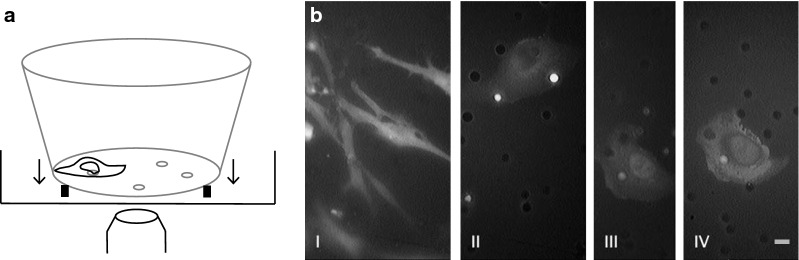
**a** Schematic of the experimental set-up. A 24-well insert of an invasion assay with MDA-MB-231 cells were placed on a shallow plastic ring into a 1-well chamber slide. This set-up allowed for confocal imaging. **b** MDA-MB-231 cells stably expressing CapG-eGFP imaged with epifluorescence in an invasion assay. I: Cancer cells not invading but remaining on top of the porous membrane are spindle shaped. Figure II-IV: Cancer cells that did invade the pores of an invasion assay showed a change in morphology and assumed an amoeboid-like cell shape. Scale bar 10 um

MDA-MB-231 cells stably expressing the GFP labeled actin-binding protein CapG were seeded into a commercially available Matrigel invasion assay with inserts comprising 8-μm pores and reconstituted basal membrane. Placing the 24-well insert onto a thin sterilized plastic ring inside a 1-well Lab-Tek chamber slide allowed for confocal live-cell imaging (Fig. 1a and “Materials and Methods” Section). As previously shown, the stable expression of CapG-eGFP did not change the number of invasive MDA-MB-231 cells compared to native MDA-MB-231 cells in this invasion assay, while CapG knockdown reduced and CapG-eGFP rescue restored invasiveness [9].

When imaged after 16 h with epifluorescence, the MDA-MB-231 cells that had not invaded the porous membrane, showed a spindle shape, while the cells that did invade the porous membrane, showed no polarization and appeared amoeboid in shape. All 25 of the 25 imaged cells that had invaded the porous membrane in three independent experiments exhibited this change of shape (Fig. 1b and Additional file 1: Movie S1). An exact quantification of the fraction of invasive MDA-MB-231 cells in the Matrigel invasion assay can be found in previously published data [9]. The Additional file 1: Movie S1 shows a fibroblast-like shaped cell on top of the porous membrane and an amoeboid-shaped cell just below it.

**Fig. 2:**
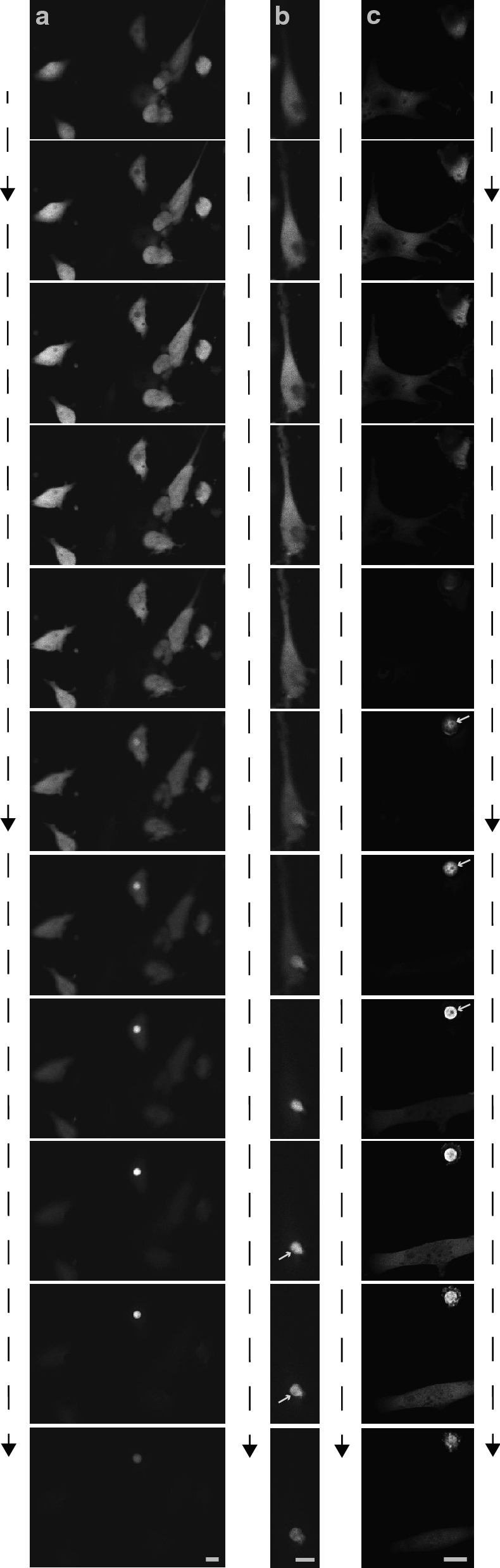
Cancer cell nucleus and adjacent cytoplasm of MDA-MB-231 cancer cells centered over a pore and moved first through the pore of an invasion assay. **a**–**c** different MDA-MB-231 cells stably expressing CapG-eGFP imaged with increasing magnification. Montage of z-stack images from top to bottom of an invasion assay. Arrowheads at dashed line indicate direction of cell invasion. Grey arrows in B and C indicate nucleoli and thereby mark the cancer cell nucleus. Scale bar 10 um

To image the cancer cells, their shape changes and nuclear positioning during invasion with higher resolution, confocal laser scanning microscopy was used. Cells were imaged 6–8 h after seeding on the porous membrane of an invasion assay. Notably, the cancer cells positioned themselves such that the cancer cell nucleus was centered over a pore. Then, the cancer cell nucleus and cytoplasmic areas in the vicinity of the cancer cell nucleus moved first through a pore of the invasion assay. Figure 2a–c show z-stacks of different cancer cells with increasing magnification. The grey arrows point at the nucleoli identifying the leading part within the pore to be the cancer cell nucleus (Fig. 2b, c). The Additional file 2: Movie S2, Additional file 3: Movie S4, and Additional file 4: Movie S4 display the entire z-stacks of the cells depicted in Fig. 2. Additional file 5: Movie S5, Additional file 6: Movie S6, and Additional file 7: Movie S7 exhibit 3-D projections which again demonstrate that the cell nucleus passes first through the pore of an invasion assay. 7 out of 7 cells, that had been imaged invading the pores of an invasion assays on three different experiment days showed the same behavior.

### Discussion

Here, I provide observational evidence that the cancer cell nucleus is leading the way during cancer cell invasion in-vitro. While most of the available studies, analyzed the cytoplasm and cytoskeleton during cell migration and cancer cell invasion, fewer and more recent studies addressed the behavior of the cell nucleus in motile cells [5]. In 3-dimensional collagen gels, the cell nucleus of dendritic cells was squeezed through constrictions by actomyosin of the trailing edge and assumed a rather passive function [7]. In a more recent publication of the Sixt group, the cell nucleus of dendritic cells was shown to be probing the microenvironment of microfluidic channels for appropriate pore sizes in order to find the path of least resistance, implying a more active role of the cell nucleus in cell migration [8]. In benign enucleated cells, specifically 3-D migration was impaired emphasizing the potential significance of the cell nucleus for cell locomotion [10]. Cancer cells with decreased lamin A content exhibited a more flexible nuclear envelope and increased invasiveness, however, more frequently nuclear envelope rupture events [11]. Nuclear envelope rupture during cancer cell migration through micrometer-sized constrictions in-vitro was repaired by the endosomal sorting complex required for transport, ESCRT machinery [12, 13]. This data from the literature and the findings presented here suggest a rather active role of the cancer cell nucleus in cancer cell invasion and metastasis.

The presented in-vitro assay captures cancer cell invasion at the intersection of 2- and 3-dimensional space locomotion. Cancer cells move on a 2-dimensional surface to locate membrane pores. Pore and basal membrane invasion are a rather confined 3-dimensional movement. Since the direction of locomotion is axial in this invasion assay, gravity may play a role in cancer cell nucleus positioning and movement. The cancer cell shape appeared amoeboid without obvious polarization after the passage through the porous membrane. However, other criteria of amoeboid locomotion were not assessed in this study including the position of the microtubules organizing center relative to the cell nucleus, the cell’s proteolytic activity and integrin density.

## Limitations

The limitations of the presented findings are their observational nature. Furthermore, the findings involve MDA-MB-231 cancer cell clones that stably expressed CapG-eGFP which needs to be considered before generalizing the results to other cell types. The study is an in-vitro study using an established cancer cell 2-/ 3-D invasion assay which may be different from in-vivo 3-dimensional cancer cell behavior.

## Supplementary information

**Additional file 1: Movie S1.** z-stack images of a spindle-shaped cell that did not invade and remained on top of the porous membrane of an invasion assay and an amoeboid-shaped cell that did invade the membrane on the underside of that porous membrane.

**Additional file 2: Movie S2.** z-stack images of Fig. 2a compiled in a movie from top down in the direction of cancer cell invasion.

**Additional file 3: Movie S3.** z-stack images of Fig. 2b compiled in a movie from top down in the direction of cancer cell invasion.

**Additional file 4: Movie S4.** z-stack images of Fig. 2c compiled in a movie from top down in the direction of cancer cell invasion.

**Additional file 5: Movie S5.** 3-D projection and 180° rotation of the cells shown in Additional file 2: Movie S2.

**Additional file 6: Movie S6.** 3-D projection and 180° rotation of the cell shown in Additional file 3: Movie S3.

**Additional file 7: Movie S7.** 3-D projection and 180° rotation of the cells shown in Additional file 4: Movie S4.

## Data Availability

Most of the raw data are presented in manuscript and additional files. Plasmid and additional raw data are available upon request.

## Abbreviations

DMEM: Dulbecco’s modified Eagle’s media
eGFP: Enhanced green fluorescent protein
FBS: Fetal bovine serum
PCR: Polymerase chain reaction
